# Synergistic Inhibition of Triple-Negative Breast Cancer by Acetylsalicylic Acid and Recombinant Human APE1/Ref-1 in a Mouse Xenograft Model

**DOI:** 10.3390/biomedicines13112767

**Published:** 2025-11-12

**Authors:** Hao Jin, Yu Ran Lee, Sungmin Kim, Eunju Choi, Ka-Young Lee, Hee Kyoung Joo, Eun-Ok Lee, Cuk-Seong Kim, Je Ryong Kim, Sang Hun Lee, Byeong Hwa Jeon

**Affiliations:** 1 Department of Physiology, College of Medicine, Chungnam National University, 266 Munhwa-ro, Jung-gu, Daejeon 35015, Republic of Korea; jinhao0508@gmail.com (H.J.); lyr0913@gmail.com (Y.R.L.); s13845@cnu.ac.kr (S.K.); cej@o.cnu.ac.kr (E.C.); pasta0901rose@gmail.com (K.-Y.L.); hkjoo79@cnu.ac.kr (H.K.J.); y21c486@naver.com (E.-O.L.); cskim@cnu.ac.kr (C.-S.K.); 2 Department of Medical Science, College of Medicine, Chungnam National University, Daejeon 35015, Republic of Korea; 3 Research Institute of Medical Sciences, College of Medicine, Chungnam National University, Daejeon 35015, Republic of Korea; 4 Department of Surgery, Chungnam National University Hospital, 282, Munhwa-ro, Jung-gu, Daejeon 35015, Republic of Korea; kimjr@cnu.ac.kr; 5 Department of Chemical and Biological Engineering, Hanbat National University, Daejeon 34158, Republic of Korea

**Keywords:** triple-negative breast cancer (TNBC), APE1/Ref-1, acetylsalicylic acid (ASA), apoptosis, MDA-MB-231

## Abstract

**Background:** Triple-negative breast cancer (TNBC) is a highly aggressive subtype with limited therapeutic options due to the lack of estrogen, progesterone, and HER2 receptors. This study investigated the synergistic anticancer effects of recombinant human apurinic/apyrimidinic endonuclease 1/redox factor-1 (rhAPE1/Ref-1) and acetylsalicylic acid (ASA), a combination that has not been previously tested in vivo. **Methods:** We treated MDA-MB-231 TNBC cells with rhAPE1/Ref-1, ASA, or their combination to assess cell viability and apoptosis in vitro. In vivo, a murine xenograft model was established to evaluate the efficacy of the combination treatment on tumor growth, tumor-specific biomarkers, and key apoptotic proteins. The safety profile of the combination therapy was also assessed by monitoring hematological parameters. **Results:** While monotherapy with either rhAPE1/Ref-1 or ASA had minimal effects, their combination significantly reduced cell viability and enhanced apoptosis in vitro by increasing DNA fragmentation. These synergistic cytotoxic effects were significantly inhibited by the receptor for advanced glycation end-products (RAGE) siRNA, suggesting that RAGE acts as an important mediator. In the xenograft model, the combination treatment suppressed tumor growth by approximately 70%, an effect comparable to paclitaxel (PTX). This was confirmed by a significant reduction in the plasma levels of TNBC biomarkers (CEA, CA27-29, and CA15-3) and increased tumor apoptosis via the upregulation of p53 and Bax and downregulation of Bcl-2. Notably, ASA, alone or combined with rhAPE1/Ref-1, induced the expression of RAGE in MDA-MB-231 tumors. In contrast to PTX, the combination of rhAPE1/Ref-1 and ASA did not cause hematological toxicity, such as anemia or thrombocytopenia. **Conclusions:** The combination of rhAPE1/Ref-1 and ASA represents a promising new therapeutic strategy for TNBC by enhancing apoptosis and significantly inhibiting tumor progression in a mouse xenograft model.

## 1. Introduction

Triple-negative breast cancer (TNBC) is an aggressive breast cancer subtype lacking estrogen receptors, progesterone receptors, and human epidermal growth factor receptor 2 [1]. This absence of key receptors limits the effectiveness of conventional hormone and targeted therapies, making TNBC particularly challenging to treat with high recurrence and metastasis risks. Therefore, developing innovative and effective treatment strategies for TNBC is crucial [2].

Apurinic/apyrimidinic endonuclease 1/redox factor-1 (APE1/Ref-1, also known as APE1) is a multifunctional protein crucial for DNA repair and redox regulation. APE1/Ref-1 overexpression in various cancers, including breast cancer, is linked to aggressive tumor phenotypes and poor prognosis, highlighting its role in cancer progression [3]. In TNBC specifically, APE1/Ref-1 has been identified as a potential prognostic marker, with high expression levels associated with lymph node involvement and disease progression [4]. Thus, targeting APE1/Ref-1 may enhance TNBC cells’ sensitivity to anticancer therapies [5].

Acetylation has emerged as a therapeutic mechanism, particularly via acetylsalicylic acid (ASA), which is known for its anti-inflammatory and anticancer properties. In addition to its well-established cardiovascular benefits [6], the anti-tumor activity of ASA is strongly supported by its ability to modulate chronic inflammation, a key driver of tumorigenesis, through cyclooxygenase inhibition [7]. Furthermore, large scale epidemiological studies showed regular low-dose ASA use with breast cancer risk [8,9,10]. Furthermore, acetylation can alter protein function, potentially revealing novel therapeutic targets. Our previous research highlighted that trichostatin A (TSA) can induce the secretion of Ac-APE1/Ref-1 [11]. This hyperacetylation reduces cell viability, promotes apoptosis, and upregulates the expression of the receptor for advanced glycation end-products (RAGE). This effect has been observed across various TNBC cell lines, including BT-549 and MDA-MB-468, suggesting that hyperacetylation could serve as a potential therapeutic strategy [12].

In TNBC xenografts, hyperacetylation has been found to trigger Ac-APE1/Ref-1 secretion into the bloodstream, where it binds to RAGE in tumor tissues. This interaction strongly inhibits tumor growth, induces apoptosis, increases RAGE expression, and elevates reactive oxygen species production. The treated xenograft tissues exhibited reduced tumor proliferation, less neovascularization, and more apoptotic bodies compared with control tumors. Additionally, the pro-apoptotic effect of Ac-APE1/Ref-1 is markedly diminished in RAGE-knockdown models, highlighting RAGE’s essential role in mediating this pathway [13]. APE1/Ref-1 is secreted in response to acetylation and binds to RAGE to initiate apoptotic cell death in TNBC, both in vitro and in vivo. Using an adenoviral vector expressing PPTLS-tagged APE1/Ref-1 (Ad-PPTLS-APE1/Ref-1) enables sustained extracellular APE1/Ref-1 expression. APE1/Ref-1 acts as both an apoptosis inducer and an inflammation modulator [14].

Although the individual effects of ASA and APE1/Ref-1 have been documented, their combined therapeutic potential for TNBC has not been reported in vivo. Therefore, in this study, we aimed to evaluate the efficacy of combining ASA with rhAPE1/Ref-1 for TNBC treatment in rodent models.

## 2. Materials and Methods

### 2.1. Materials

Acetyl-salicylic acid (ASA) and paclitaxel (PTX) were purchased from Sigma Aldrich (St. Louis, MO, USA). Antibodies were used against Bcl-2 (NB100-56098, 1:1000, Novus Biologicals, Centennial, CO, USA), Bax (NBP1-28566, 1:1000, Novus Biologicals), RAGE (SC-365154, 1:1000, Santa Cruz Biotechnology, Dallas, TX, USA), p53 (SC-126, 1:1000, Santa Cruz Biotechnology), rhAPE1/Ref-1 (MR-RPAPE-010, MediRedox), and GAPDH (SC-32233, 1:1000, Santa Cruz Biotechnology). RAGE siRNA (sc-36373) and its control siRNA were purchased from Santa Cruz Biotechnology. Endotoxin-free recombinant human APE1/Ref-1 protein (rhAPE1/Ref-1; #MR-EAPE-100) was purchased from MediRedox Inc. (Daejeon, Republic of Korea). ASA was dissolved to a 10 mM concentration in distilled water, with pH adjusted to 7.4 using sodium hydroxide. rhAPE1/Ref-1 was reconstituted in phosphate-buffered saline (PBS; 137 mM NaCl, 10 mM phosphate, 2.7 mM KCl, pH 7.4).

### 2.2. Cell Culture

The human breast adenocarcinoma cell line, MDA-MB-231 (ATCC^®®^ HTB-26™), was obtained from the American Type Culture Collection (Manassas, VA, USA). Luciferase-expressing MDA-MB-231 cells (MDA-MB-231-Red-FLuc CVCL-5J05) were purchased from PerkinElmer (Waltham, MA, USA). Both cell lines were grown as monolayers in 75 mL tissue culture flasks at 37 °C in a humidified 5% CO_2_ incubator. MDA-MB-231 and MDA-MB-231-FLuc cells were cultured in RPMI 1640 medium (Gibco™, Thermo Fisher Scientific, Waltham, MA, USA) supplemented with 10% heat-inactivated fetal bovine serum and 1% antibiotics (Gibco™, Thermo Fisher Scientific), respectively.

### 2.3. Gene Silencing of RAGE

RAGE expression was knocked down using RNA interference (RNAi). Briefly, MDA-MB-231 cells were transfected with either a control siRNA or a specific siRNA targeting RAGE using Lipofectamine RNAiMAX transfection reagent (Invitrogen, Waltham, MA, USA) according to the manufacturer’s instructions.

### 2.4. Immunofluorescence Staining for Apoptosis Analysis

Apoptosis in MDA-MB-231 cells was assessed using the Annexin V-FITC Apoptosis Detection Kit (Cat. No. ab14085, Abcam, Cambridge, UK) according to the manufacturer’s guidelines. MDA-MB-231 cells (1 × 10^5^ cells/coverslip) were seeded on coverslips and treated with ASA and/or rhAEP1/Ref-1. After incubation for 24 h, the cells were stained with Annexin V-FITC and propidium iodide (PI) for 10 min. Thereafter, the cells were fixed in 2% formaldehyde, and their nuclei were counterstained with 4′,6-diamidino-2-phenylindole (DAPI). Images were captured using a fluorescence microscope (Axiocam 305, Carl Zeiss Microscopy, Jena, Germany).

### 2.5. Cell Viability and DNA Fragmentation Assay

MDA-MB-231 cells were cultured in 96-well plates and treated for 24 h with rhAPE1/Ref-1 or ASA. To measure viability, 10 μL of MTT reagent (5 mg/mL) was added and incubated for 2 h at 37 °C. Formazan crystals were dissolved with 100 μL of DMSO, and the absorbance was read at 600 nm using a GloMax Discover microplate reader (Promega, Madison, WI, USA). Quantification of DNA fragmentation was performed using a sandwich-type ELISA, which detects cytoplasmic histone-associated DNA fragments generated by endonuclease activation. DNA fragmentation following treatment was evaluated using the Cell Death Detection ELISA kit (Roche Life Science, Basel, Switzerland) in accordance with the manufacturer’s instructions.

### 2.6. Animal Experiments

Seven-week-old female BALB/c nude mice were purchased from Doo-Yeol Biotech (Seoul, Republic of Korea). The mice were housed at 24 °C with a 12 h day/night cycle under specific pathogen-free conditions, with ad libitum access to γ-ray-irradiated laboratory rodent diet (Purina, Seongnam, Republic of Korea) and autoclaved water. All experiments adhered to Chungnam National University’s animal care guidelines and were approved by the Ethics Committee of Animal Experimentation (Approval no. CNUH-020-A0026). MDA-MB-231-FLuc cell lines constitutively expressing luciferase (MDA-MB-231-Red-FLuc Bioware^®®^ Brite CVCL-5J05) were used to generate orthotopic xenografts. The mice were anesthetized, and a 5 mm skin incision was made below the fourth nipple. A total of 1.5 × 10^6^ MDA-MB-231-FLuc cells mixed in 100 µL Matrigel (Corning, NY, USA) was injected into the left inguinal mammary fat pad of 8-week-old female BALB/c nude mice. To assess the effects of rhAPE1/Ref-1 combined with ASA, the MDA-MB-231-FL-injected mice were randomly divided into four groups: control (TNBC alone), rhAPE1/Ref-1 (2.5 mg/kg, three times/week), ASA (50 mg/kg three times/week), and ASA (50 mg/kg) + rhAPE1/Ref-1 (2.5 mg/kg). A PTX group (10 mg/kg, once/week via intraperitoneal injection) was also included until the study endpoint, with 5–7 mice per group and treated for 6 weeks. For the removal of animals from the experiment, humane endpoints were strictly observed, including monitoring for significant weight loss (>20%). At the conclusion of the study, all mice were euthanized using CO_2_ asphyxiation, which is an approved standard method. Bioluminescence imaging of tumors using the IVIS Spectrum in vivo imaging system (Perkin Elmer, Shelton, CT, USA) was performed biweekly post-xenografting to confirm the presence of live tumor cells. Tumor size was measured twice weekly using a digital caliper until the endpoint, when bioluminescence imaging of tumors was repeated before tissue collection. At the endpoint, tumor tissues were harvested and used for histological and immunoblot analyses. All animal experiments were repeated at least twice with similar results.

### 2.7. Western Blotting

Tumor tissue samples were homogenized and lysed in RIPA buffer (150 mM NaCl, 1.0% NP-40, 0.5% sodium deoxycholate, 0.1% sodium dodecyl sulfate (SDS), 50 mM Tris-HCl, pH 8.0) supplemented with a protease inhibitor cocktail (Cat. No. 11836170001, Roche, Basel, Switzerland). In total, 30 µg of proteins per sample was separated using 10% sodium dodecyl sulfate–polyacrylamide gel electrophoresis. Thereafter, proteins were transferred to a polyvinylidene difluoride (PVDF) membrane using wet transfer at 100 V for 1 h. The membrane was blocked with 5% non-fat dry milk in Tris-buffered saline with Tween 20 (TBST) (20 mM Tris-HCl, 150 mM NaCl, 0.1% Tween 20, pH 7.6) for 1 h at 21 °C. Thereafter, the membrane was incubated overnight at 4 °C with the primary antibodies against Bax, Bcl-2, GAPDH, p53, APE1/Ref-1 (MR-PAAPE, MediRedox, 1:1000), and RAGE. After washing three times with TBST, the membrane was incubated with HRP-conjugated secondary antibodies, goat anti-rabbit IgG-HRP (Cat. No. 31460, 1:5000, Thermo Fisher Scientific), and goat anti-mouse IgG-HRP (Cat. No. 31430, 1:5000, Thermo Fisher Scientific) for 1 h at room temperature. Following three additional washes with TBST, protein bands were visualized via enhanced chemiluminescence and quantified using ImageJ software (version Fiji 2.3.0), with normalization to GAPDH.

### 2.8. Tumor-Specific Biomarker Analysis

Serum samples were diluted 1:10 for the subsequent ELISA using the following kits: Mouse Carbohydrate Antigen 15-3 (CA15-3, Cat. No. MBS160258, MyBioSource, San Diego, CA, USA), Mouse Cancer Antigen 27-29 (Cat. No. 2613555, MyBioSource), and Mouse Carcinoembryonic Antigen (Cat. No. LS-F5042, LSBio, Seattle, WA, USA), as per the manufacturer’s instructions. The ELISA procedure followed the manufacturer’s protocol, including instructions on incubation times, incubation temperatures, and washing steps. 3,3′,5,5′-teramethylbenzidine substrate was used for colorimetric detection, and the absorbance was measured at 450 nm using a Promega GloMax^®®^ Discover System plate reader (Promega, Madison, WI, USA). Biomarker concentrations were calculated using a standard curve and reported in ng/mL.

### 2.9. Complete Blood Counts

The mice were fasted for 16 h overnight. Blood samples were collected via heart puncture following anesthesia with a ketamine (80 mg/kg) and xylazine mixture (10 mg/kg). Thereafter, the blood samples were transferred to EDTA tubes for complete blood cell count analysis, performed using an XN-V hematology analyzer (Sysmex, Kobe, Japan).

### 2.10. Immunohistochemistry

Tissues were fixed in a 10% neutral-buffered formalin solution for 24 h, embedded in paraffin blocks, sectioned (4 μm thick), and subjected to hematoxylin and eosin (H&E) staining. The sections were subsequently incubated overnight at 4 °C with primary anti-RAGE antibodies. Thereafter, HRP-conjugated anti-rabbit or anti-mouse IgG was applied for 60 min at room temperature. Color development was induced by incubating the sections with 3,3′-diaminobenzidine (DakoCytomation, Santa Clara, CA, USA). Then, the sections were counterstained with H&E and examined under a Motic microscope (Motic, Richmond, BC, Canada) at 4× to 40× magnification.

### 2.11. In Vivo Optical Imaging

Bioluminescence imaging was performed using an in vivo imaging system (IVIS) consisting of a Lumina XRMS instrument (PerkinElmer, Shelton, CT, USA). The animals were anesthetized via inhalation of 2.5% isoflurane and received intraperitoneal injections of RediJect D-luciferin potassium salt (150 mg/kg). Images were captured and analyzed using Living Image software (version 4.7.4, Caliper Life Sciences, Street Waltham, MA, USA). A luminescent camera captured images every 30 s using medium binning, 1 f/stop, a blocked excitation filter, and an open emission filter. Photon emissions from live mice were recorded as relative luminescence units (proportional to the number of photons detected in the pixel) and as photons per s/[cm^2^·steradian (sr)].

### 2.12. TUNEL Assay

Apoptotic cells in TNBC tissues were detected using the ApopTag^®®^ Peroxidase in situ Apoptosis Detection Kit (Cat. No. S7100, EMD Millipore, Billerica, MA, USA) according to the manufacturer’s instructions with minor modifications. TNBC tumor tissues were fixed in 10% neutral-buffered formalin, trimmed into 2–3 mm thick pieces, and processed using an automated tissue processor for paraffin embedding. The paraffin blocks were sectioned at a 4 μm thickness and mounted on silane-coated slides. The sections were deparaffinized in xylene, rehydrated using a graded ethanol series, and rinsed in distilled water. For permeabilization, the slides were incubated with Proteinase K for 10 min at room temperature, followed by washing with PBS. Endogenous peroxidase activity was quenched using Peroxide Blocking Reagent (DAKO, Santa Clara, CA, USA) for 10 min at room temperature. Thereafter, the slides were incubated with Equilibration Buffer for 1 min and subsequently with TdT enzyme in a humidified chamber for 1 h at room temperature. The reaction was stopped by washing with Stop/Wash Buffer for 10 min. The slides were incubated with Anti-Digoxigenin-Peroxidase Conjugate for 30 min at room temperature. Color development was performed using DAB substrate, and nuclei were counterstained with Mayer’s hematoxylin. After dehydration and clearing, the slides were mounted with coverslips. TUNEL-positive nuclei were visualized under a light microscope as brown-stained cells, indicating DNA fragmentation and apoptosis.

### 2.13. Statistical Analyses

Group means were compared using unpaired t-test or one-way analysis of variance (ANOVA), followed by Dunnett’s multiple comparison test, with GraphPad Prism version 10 (GraphPad Software, Inc., San Diego, CA, USA). For all analyses, *p* < 0.05 was considered statistically significant.

## 3. Results

### 3.1. Combined Treatment with ASA and rhAPE1/Ref-1 Enhances Apoptosis in MDA-MB-231 Cells In Vitro

**Figure 1:**
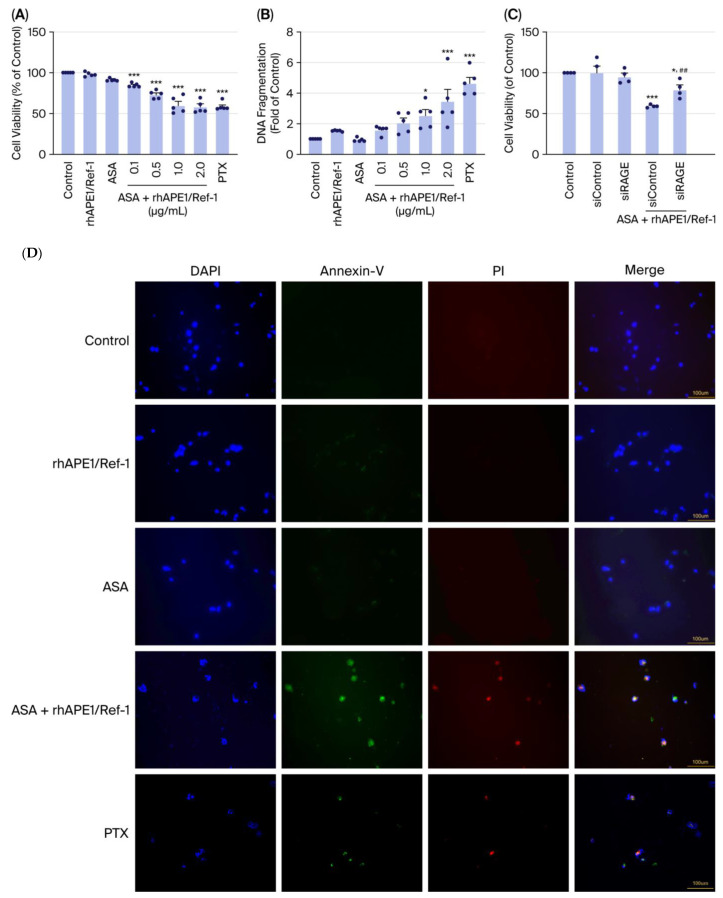
Combined treatment with acetylsalicylic acid (ASA) and recombinant human APE1/Ref-1 (rhAPE1/Ref-1) enhances apoptosis in MDA-MB-231 cells. (**A**) MDA-MB-231 cell viability following treatment with various concentrations of rhAPE1/Ref-1 (0.1~2 μg/mL) in the presence or absence of ASA (1 mM). Cell viability is expressed as a percentage relative to the control group. Data are presented as mean ± SE. (**B**) DNA fragmentation in MDA-MB-231 cells treated with rhAPE1/Ref-1 (0.1~2 μg/mL) in the presence or absence of ASA (1 mM). DNA fragmentation is expressed as a fold change relative to the control group. Data are presented as mean ± SE. (**C**) The effect of gene silencing of receptor for advanced glycation end-products (RAGE) using small interference RAGE (siRAGE) on reduced cell viability via co-treatment with ASA and rhAPE1/Ref-1 in MDA-MB-231 cells. Cell viability is expressed as a percentage relative to the control group. Data are presented as mean ± SE. * *p* < 0.05, *** *p* < 0.005 vs. control and ^##^ *p* < 0.01 vs. siControl with ASA+rhAPE1/Ref-1. (**D**) Representative immunofluorescence images of MDA-MB-231 cells treated with the indicated rhAPE1/Ref-1 (2 μg/mL), ASA (1 mM), or paclitaxel (PTX, 5 μg/mL) for 24 h. Early apoptosis was detected via Annexin V staining (green), and late apoptosis/necrosis was detected via propidium iodide (PI) staining (red). The cell nuclei were counterstained with DAPI (blue). Pink indicates the merged signal. Scale bar = 100 μm.

To investigate the in vitro interaction between rhAPE1/Ref-1 and ASA in MDA-MB-231 breast cancer cells, we first assessed cell viability and DNA fragmentation. Importantly, treatment with either rhAPE1/Ref-1 or ASA alone did not significantly alter cell viability compared with the untreated control. However, combined treatment with rhAPE1/Ref-1 (0.1~2.0 µg/mL) and ASA markedly reduced cell viability in a dose-dependent manner, indicating a synergistic cytotoxic effect. Specifically, co-treatment with rhAPE1/Ref-1 (2.0 µg/mL) and ASA reduced cell viability to approximately 60% (Figure 1A). Moreover, DNA fragmentation did not increase in cells treated with either ASA or rhAPE1/Ref-1 alone. However, combined treatment with both rhAPE1/Ref-1 (1.0~2.0 µg/mL) and ASA significantly increased DNA fragmentation. Notably, compared with the control group, rhAPE1/Ref-1 (2.0 µg/mL) and ASA co-treatment resulted in an approximately four-fold increase in DNA fragmentation, suggesting that the combined treatment induces cell death via enhanced DNA damage (Figure 1B). Treatment with paclitaxel (PTX), which served as a positive control, also resulted in a decrease in cell viability and a corresponding increase in DNA fragmentation in MDA-MB-231 cells (Figure 1A,B). To investigate the role of RAGE in this synergistic effect, we performed an MTT assay after silencing RAGE expression. Crucially, the reduction in cell viability induced by rhAPE1/Ref-1 (2.0 µg/mL) + ASA was significantly inhibited when cells were pre-treated with siRAGE relative to siControl (Figure 1C). This strongly suggests that RAGE is an important mediator in the cell death induced by the combination of rhAPE1/Ref-1 and ASA. The induction of apoptosis was confirmed by Annexin-V and PI immunofluorescence imaging (Figure 1D). In the combination group, immunofluorescence imaging of MDA-MB-231 cells co-treated with rhAPE1/Ref-1 and ASA showed an increase in Annexin V and PI fluorescence intensities relative to all other single-treatment conditions (Figure 1D). As a positive control, PTX treatment also induced increased Annexin V and PI fluorescence intensities (Figure 1D). This overall observation confirmed that the combined application of rhAPE1/Ref-1 and ASA induces apoptosis in MDA-MB-231 cells.

### 3.2. Combined Effects of ASA and rhAPE1/Ref-1 on Tumorigenesis in an MDA-MB-231 Xenograft Model

**Figure 2:**
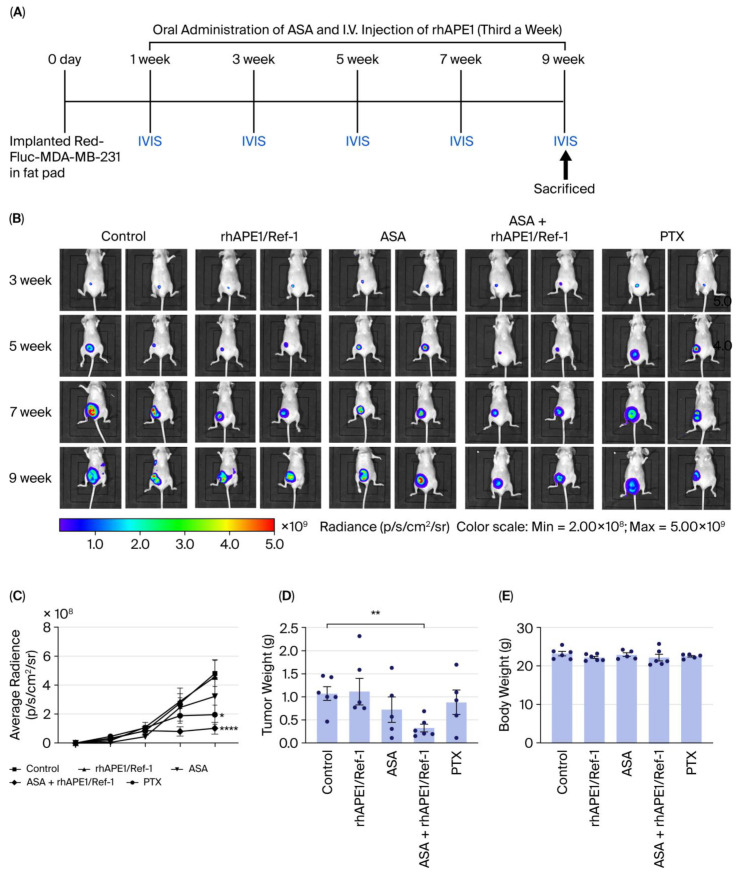
Synergistic effects of ASA and rhAPE1/Ref-1 on tumorigenesis in a TNBC mouse model. (**A**) Treatment schedule depicting experimental groups of TNBC tumor-bearing mice: control (TNBC alone), rhAPE1/Ref-1, ASA, ASA + rhAPE1/Ref-1, and paclitaxel (PTX). Red-Fluc-MDA-MB-231 cells were implanted in the fat pads. (**B**) In vivo IVIS imaging of TNBC tumor-bearing mice at weeks 3, 5, 7, and 9. The images display spatial distribution and intensity of luminescence, representing tumor burden in each treatment group: control, rhAPE1/Ref-1, ASA, ASA + rhAPE1/Ref-1, and PTX. The color scale indicates radiance in photons/s/cm^2^/steradian. (**C**) Quantitative analysis of IVIS signal intensity over time in TNBC tumor-bearing mice treated with control, rhAPE1/Ref-1, ASA, ASA + rhAPE1/Ref-1, or PTX. Data are presented as mean ± SE. * *p* < 0.05 vs. control; **** *p* < 0.0001 vs. control. (**D**) Tumor weight (g) at the endpoint in TNBC tumor-bearing mice. Data are presented as mean ± SE. ** *p* < 0.01 vs. control. (**E**) Body weight (g) at the endpoint in TNBC tumor-bearing mice. Data are presented as mean ± SE, with *n* = 6 mice per group.

After confirming that treatment with both rhAPE1/Ref-1 and ASA induced apoptosis in MDA-MB-231 cells in vitro, their combined ability to suppress tumor growth in vivo was examined using a murine TNBC model. Red-Fluc-MDA-MB-231 cells were implanted into the mammary fat pads of mice, and tumor growth was monitored over 9 weeks. The mice were randomized into five treatment groups (Figure 2A): TNBC control, rhAPE1/Ref-1, ASA, ASA + rhAPE1/Ref-1, and PTX. ASA was administered orally, while rhAPE1/Ref-1 was administered intravenously three times a week. Bi-weekly IVIS imaging (Figure 2B) demonstrated progressive tumor growth in the control group. Notably, although monotherapy with either rhAPE1/Ref-1 or ASA did not significantly inhibit tumor growth, their combination substantially suppressed tumor growth at a rate comparable to the PTX treatment (Figure 2C).

Specifically, the ASA + rhAPE1/Ref-1 combination resulted in a significant reduction of approximately 70% in tumor weight compared with the TNBC control group (Figure 2D). Notably, body weight analysis (Figure 2E) revealed no significant changes across groups, suggesting good tolerability of the combination therapy. These in vivo findings, consistent with the previously observed apoptotic effects in vitro, highlight the promising synergistic potential of combining rhAPE1/Ref-1 and ASA as a therapeutic approach for TNBC.

### 3.3. Combined Treatment of rhAPE1/Ref-1 with ASA Reduces TNBC Tumor-Specific Biomarkers In Vivo

**Figure 3:**
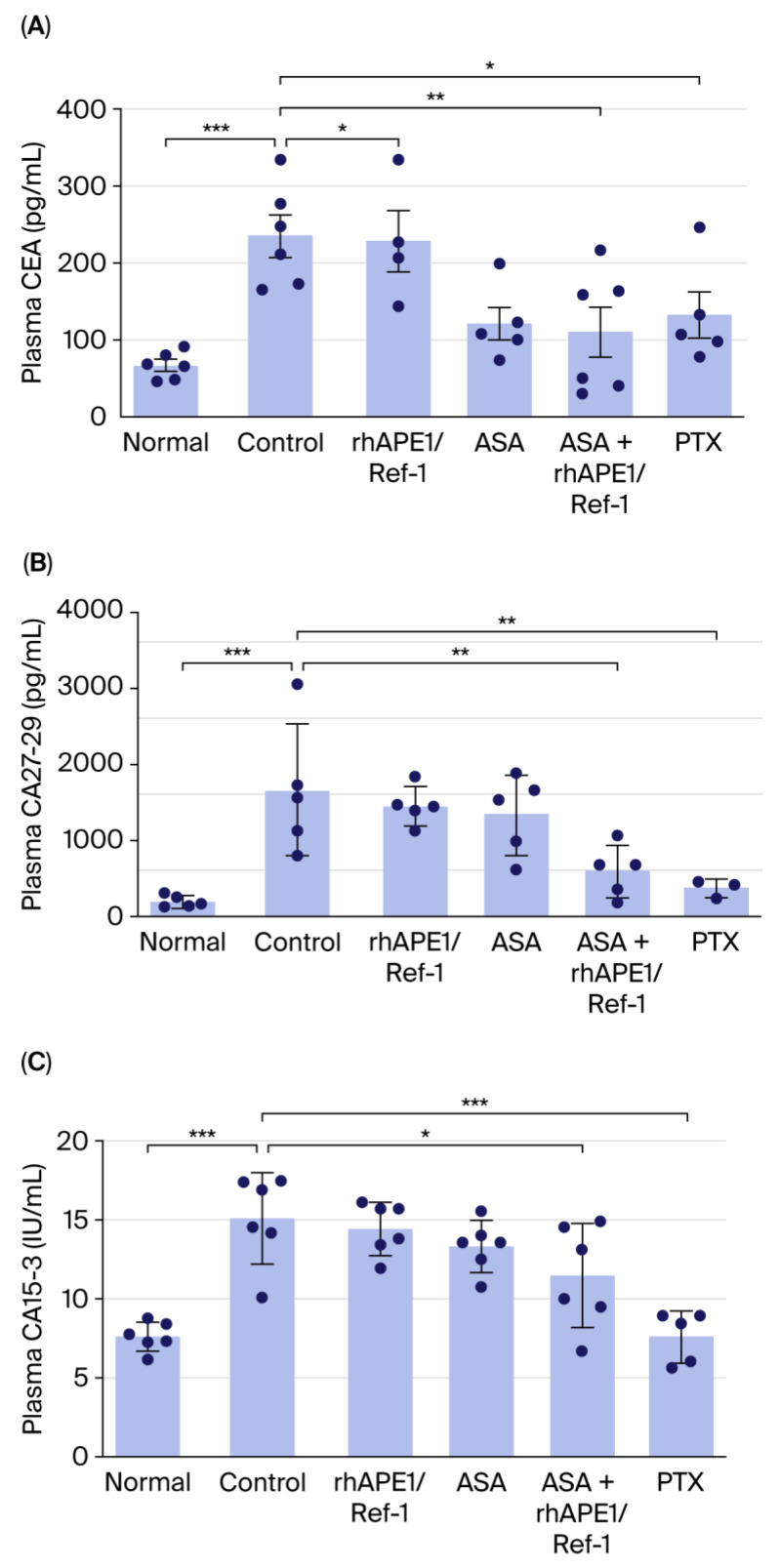
Combination treatment of rhAPE1/Ref-1 and ASA reduces TNBC tumor-specific biomarkers in vivo. (**A**) Plasma levels of CEA (pg/mL) in normal mice and TNBC tumor-bearing mice treated with control (TNBC alone), rhAPE1/Ref-1, ASA, ASA + rhAPE1/Ref-1, or PTX. Data (*n* = 4–6) are presented as mean ± SE. * *p* < 0.05, ** *p* < 0.01, and *** *p* < 0.001 vs. control. (**B**) Plasma levels of CA27-29 (pg/mL) in normal mice and TNBC tumor-bearing mice. Data (*n* = 3–6) are presented as mean ± SE. ** *p* < 0.01 and *** *p* < 0.001 vs. control. (**C**) Plasma levels of CA15-3 (IU/mL) in normal mice and TNBC tumor-bearing mice. Data (*n* = 6) are presented as mean ± SE. * *p* < 0.05 and *** *p* < 0.001 vs. control.

To determine whether rhAPE1/Ref-1 or ASA affects TNBC tumor-specific biomarkers, changes in CEA, CA27-29, and CA15-3 plasma levels in the normal, TNBC control, rhAPE1/Ref-1-treated, ASA-treated, rhAPE1/Ref-1+ ASA-treated, and PTX-treated groups were examined. As shown in Figure 3A, plasma CEA levels were elevated in the TNBC xenograft group compared with those in the normal group (80 pg/mL for normal vs. 230 pg/mL for TNBC). rhAPE1/Ref-1 treatment alone did not significantly alter CEA levels. However, ASA, rhAPE1/Ref-1 + ASA, and PTX treatments significantly suppressed CEA levels. Similarly, Figure 3B illustrates that plasma CA27-29 levels increased in the TNBC xenograft group (100 pg/mL for normal vs. 1700 pg/mL for TNBC). Neither rhAPE1/Ref-1 nor ASA monotherapy significantly affected CA27-29 levels. In contrast, rhAPE1/Ref-1 + ASA and PTX treatments significantly reduced CA27-29 levels. Furthermore, Figure 3C demonstrates that plasma CA15-3 levels were also elevated in the TNBC xenograft group (7 IU/mL for normal vs. 15 IU/mL for TNBC). Consistent with the previous findings, rhAPE1/Ref-1 and ASA alone did not significantly change CA15-3 levels, whereas rhAPE1/Ref-1 + ASA and PTX treatments significantly decreased CA15-3 levels.

Collectively, although rhAPE1/Ref-1 or ASA alone did not significantly alter tumor biomarker levels, the combination therapy of rhAPE1/Ref-1 with ASA significantly reduced plasma CEA, CA27-29, and CA15-3 levels. These findings suggest that the combination therapy of rhAPE1/Ref-1 with ASA may have a synergistic effect in inhibiting tumor growth and reducing TNBC tumor-specific biomarker levels.

### 3.4. Combination Treatment of rhAPE1/Ref-1 and ASA Increases Tumor Apoptosis in TNBC In Vivo

**Figure 4:**
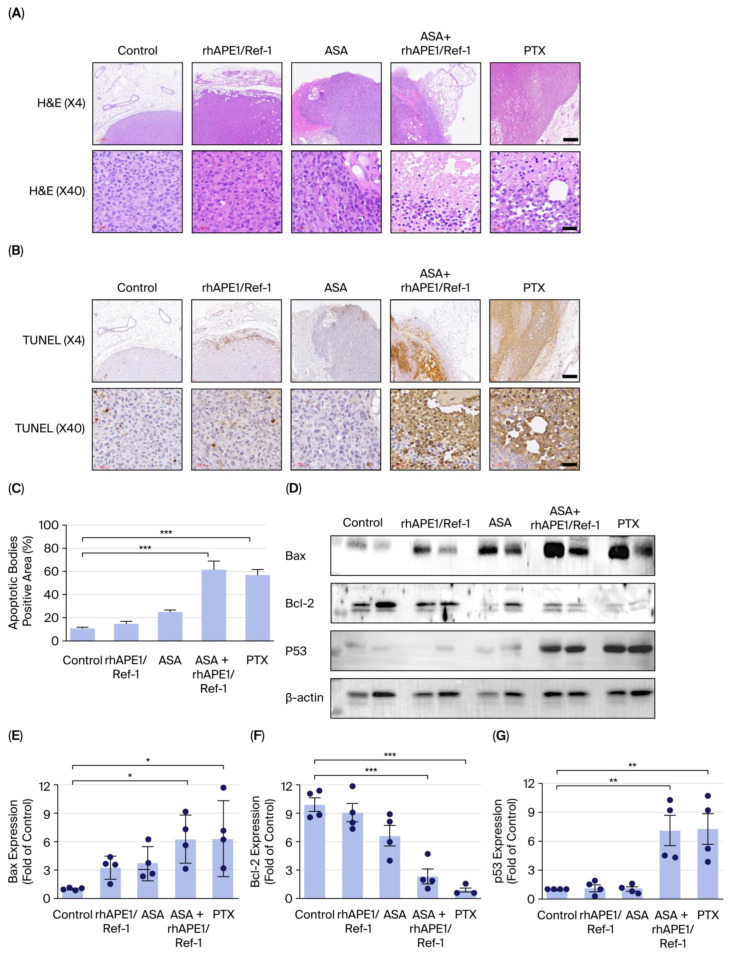
Effects of rhAPE1/Ref-1 and ASA on tumor cell apoptosis in TNBC xenograft tumors. (**A**) Representative images of hematoxylin and eosin (H&E)-stained tumor sections from mice treated with control (TNBC alone), rhAPE1/Ref-1, ASA, ASA + rhAPE1, or PTX. The upper panels (×4) show the overall tumor architecture, while the lower panels (×40) present magnified views. Scale bar = 300 μm or 60 μm. (**B**) Representative images of TUNEL assay staining, detecting DNA fragmentation associated with apoptosis. Tumors treated with the combination therapy show increased TUNEL-positive cells (brown). The upper panels (×4) provide an overview of apoptotic regions, and the lower panels (×40) show magnified views. Brown staining indicates apoptotic cells. Scale bar = 300 μm or 60 μm. (**C**) TUNEL-positive cell quantification, confirming a significant increase in apoptosis under the combination therapy compared with the control (*** *p* < 0.001). Data are presented as mean ± SE. (**D**) Representative Western blots showing Bax, Bcl-2, and p53 expression in TNBC-treated mice. (**E**) Quantification of BAX expression under indicated conditions. All values represent the mean ± SE. *n* = 4. * *p* < 0.05 vs. control. (**F**) Quantification of Bcl-2 expression under indicated conditions. All values represent the mean ± SE. *n* = 4. *** *p* < 0.001 vs. control. (**G**) Quantification of p53 expression under indicated conditions. All values represent the mean ± SE, *n =* 4. ** *p* < 0.01 vs. control. Statistical differences were determined using one-way ANOVA with Dunnett’s test for multiple comparisons.

To investigate the effects of the rhAPE1/Ref-1 and ASA combination treatment on tumor apoptosis in TNBC in vivo, the TUNEL assay for detecting DNA fragmentation, which is a hallmark of apoptosis, was performed on tumor tissues from TNBC xenograft mice treated with TNBC control, rhAPE1/Ref-1, ASA, ASA + rhAPE1/Ref-1, or PTX. Hematoxylin and eosin (H&E) staining revealed that the TNBC control group exhibited a dense, uniform tumor architecture with minimal signs of necrosis. The rhAPE1/Ref-1 and ASA monotherapy groups showed minor changes in cellular density and tumor integrity. In contrast, the ASA + rhAPE1/Ref-1 and PTX-treated groups displayed significant areas of reduced cellularity, indicating increased tumor cell death. At higher magnification (×40), the ASA + rhAPE1/Ref-1 and PTX-treated groups showed cellular debris and disrupted tissue structure, further confirming the increased tumor cell death (Figure 4A). In the TUNEL assay, minimal levels (7–10%) of positive staining were found in the TNBC control group. The TNBC tumor tissues treated with rhAPE1/Ref-1 or ASA alone showed only a marginal increase in positive staining in the TUNEL assay. In contrast, the ASA + rhAPE1/Ref-1 and PTX-treated groups displayed a substantial increase in TUNEL-positive cells throughout the tumor tissues, indicating significant apoptosis induction (Figure 4B). Qualitative assessment of TUNEL-stained sections revealed a marked increase in apoptotic cells in the ASA+rhAPE1/Ref-1 and PTX-treated groups compared with the TNBC control, rhAPE1/Ref-1, and ASA monotherapy groups (Figure 4C). These TUNEL assay results suggest that the combination of ASA+rhAPE1/Ref-1 and PTX effectively induces apoptosis in TNBC xenografts, significantly increasing tumor cell death.

To further elucidate the underlying apoptotic mechanisms, we analyzed the expression of key apoptosis-regulating proteins, including p53, Bax, and Bcl-2, via Western blotting (Figure 4D). In the ASA + rhAPE1/Ref-1 and PTX-treated groups, the pro-apoptotic proteins Bax (Figure 4E) and p53 (Figure 4G) were markedly upregulated, whereas the anti-apoptotic protein Bcl-2 (Figure 4F) was significantly downregulated. In contrast, the monotherapies had a minimal impact on the levels of these proteins compared with the control. These molecular findings are consistent with the TUNEL staining results, collectively indicating that combined administration of ASA and rhAPE1/Ref-1 enhances apoptosis in TNBC xenografts by activating the p53/Bax pathway and suppressing Bcl-2 expression.

### 3.5. ASA Induces RAGE Expression in TNBC Xenograft Tumors

RAGE is suggested as a potential target of acetylated APE1/Ref-1 [12,15]. This study investigated the impact of ASA on RAGE expression in an in vivo TNBC model. Immunohistochemistry and Western blotting were performed on tumor tissues from TNBC xenograft mice treated with control, rhAPE1/Ref-1, ASA, ASA + rhAPE1/Ref-1, or PTX.

**Figure 5:**
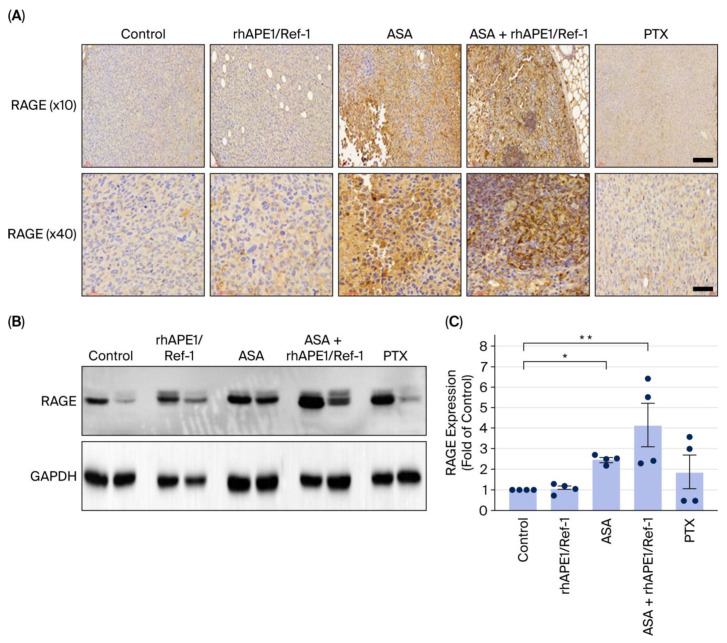
ASA increases RAGE expression in TNBC xenograft mice. (**A**) Representative immunohistochemical staining of RAGE expression in TNBC xenograft mice treated with control (TNBC alone), rhAPE1/Ref-1, ASA, ASA + rhAPE1, or PTX. The upper panels (×10) show the overall tumor architecture, while the lower panels (×40) present magnified views. Scale bar = 100 μm or 60 μm. (**B**) Representative image of Western blot analysis of RAGE protein expression in the tumor regions of TNBC xenograft mice under different conditions. GAPDH was used as a loading control. (**C**) Quantification of RAGE protein expression from the Western blot in (**B**). Data (*n* = 4) are presented as mean ± SE. * *p* < 0.05 and ** *p* < 0.001 vs. control.

Immunohistochemical staining revealed low RAGE expression in the TNBC control and rhAPE1/Ref-1-treated groups. In contrast, ASA treatment significantly increased cytoplasmic and/or nuclear RAGE staining intensity in TNBC tumors. A higher magnification (×40) revealed a more pronounced accumulation of RAGE within tumor cells in the ASA-treated group (Figure 5A). Western blot analysis (Figure 5B) showed that RAGE protein levels significantly increased with ASA treatment alone. This increase was even more pronounced when ASA was combined with rhAPE1/Ref-1, especially when compared with the control group (Figure 5C). These results indicate that ASA enhances RAGE expression in TNBC tumors.

### 3.6. Hematological Parameters of TNBC Xenograft Mice

To evaluate immunosuppression and systemic inflammation, hematological parameters were analyzed and compared across groups. The normal group consisted of mice that did not receive a TNBC xenograft. In contrast, all other groups (TNBC control, rhAPE1/Ref-1, ASA, ASA+rhAPE1/Ref-1, and PTX) were composed of mice implanted with TNBC xenografts and treated with various compounds. As shown in Table 1, in mice implanted with TNBC xenografts, the neutrophil percentage significantly increased whereas the lymphocyte percentage significantly decreased, resulting in an elevated neutrophil-to-lymphocyte ratio (NLR) (1.96 versus 0.65 in the normal group). This suggests systemic inflammation and immunosuppression in vivo. However, the ASA and rhAPE1/Ref-1 combination treatment significantly increased the lymphocyte percentage (45.2% versus 30.2% in the control group, *p* < 0.01) and significantly decreased NLR (0.89 versus 1.96 in the control group, *p* < 0.05), indicating potential mitigation of TNBC-induced immunosuppression and systemic inflammation. Interestingly, PTX treatment significantly exacerbated anemia, causing severe decreases in red blood cell count, hemoglobin, and hematocrit and inducing severe thrombocytopenia (low platelet count). While the white blood cell count remained within the normal range, the combined decreases in red blood cells and platelets indicated substantial hematological toxicity in the PTX treatment group. Notably, the combination treatment of ASA and rhAPE1/Ref-1 did not significantly affect red blood cell, hemoglobin, hematocrit, and platelet counts, suggesting its potential as a safer alternative to PTX by avoiding hematological toxicity.

## 4. Discussion

In this study, we evaluated the efficacy of combining ASA with rhAPE1/Ref-1 for TNBC treatment in rodent models. Our findings show that the ASA and rhAPE1/Ref-1 combination treatment synergistically suppresses TNBC growth both in vitro and in vivo. In vitro, the combination therapy significantly reduced MDA-MB-231 cell viability and induced DNA fragmentation, suggesting enhanced apoptosis. In vivo, the combination therapy led to a 70% reduction in tumor weight compared with the TNBC control.

TNBC remains a clinical challenge owing to its aggressive nature, lack of targeted therapies, and propensity for metastasis. Although individual agents such as the protein deacetylase inhibitor trichostatin A (TSA), secreted APE1/Ref-1 [12], PPT-LS APE1/Ref-1 [14], and acetylated recombinant human APE1/Ref-1 (Ac-rhAPE1/Ref-1) [13] have shown promise in preclinical studies, combining ASA with rhAPE1/Ref-1 remains underexplored. In the present study, we demonstrated, for the first time, the potent synergistic antitumor efficacy of combining ASA with the rhAPE1/Ref-1 protein itself using a TNBC xenograft model. This study represents a crucial in vivo validation of the effects of this therapeutic protein agent, underscoring its potential for clinical application.

ASA, widely known as aspirin, is a common non-steroidal anti-inflammatory drug and has demonstrated pleiotropic effects, including anti-inflammatory, anti-platelet, and anticancer properties [16]. Specifically, in breast cancer, ASA suppresses tumor growth and metastasis and improves distant metastasis-free survival and disease-free survival in TNBC populations [17,18]. However, high-dose ASA induces non-specific toxicity, including gastrointestinal bleeding due to the loss of prostaglandin E_2_ (PGE_2_), gastric mucosa cytoprotection [19], and reduced renal sodium excretion [20]. Therefore, the use of clinically established low-dose ASA [21] in this study offers a unique opportunity to explore its potential in combination therapy while minimizing adverse effects. Combining low-dose ASA with rhAPE1/Ref-1 offers promise for overcoming the limitations of single-target therapies, facilitating this TNBC therapy toward clinical application.

In vitro, ASA and rhAPE1/Ref-1 synergistically reduced MDA-MB-231 cell viability and MDA-MB-468 cells (Supplementary Figure S1) compared with the single-agent treatments. These results support the combination therapy’s therapeutic potential for TNBC. Therefore, we proceeded with animal studies using an MDA-MB-231 cell-derived xenograft model to validate these in vitro findings and assess the translational potential of this combination therapy.

To establish a low-dose ASA regimen comparable to that used in humans, body surface area-based scaling was applied. Mice (20–25 g) received 1 mg ASA intraperitoneally (40–50 mg/kg) three times a week. Using Km conversion factors (mouse = 3; human = 37), the human equivalent dose (HED) was determined to be 3.24–4.05 mg/kg. This HED translates to 195–243 mg/dose for a 60 kg adult, resulting in a total weekly dose of 584–729 mg [22]. Crucially, this total dose aligns with or slightly exceeds the standard low-dose ASA range in humans (75–100 mg/d). Thus, the mouse dose used in this study approximated a clinically relevant human low-dose ASA regimen.

In vivo bioluminescence imaging and tumor weight measurements revealed that the ASA and rhAPE1/Ref-1 combination therapy significantly suppressed TNBC tumor growth. Specifically, the combination therapy reduced tumor weight by approximately 70% compared with the TNBC control group (*p* < 0.05). This potent antitumor effect highlights the synergistic action of ASA and rhAPE1/Ref-1.

The combination of ASA and rhAPE1/Ref-1 significantly reduced the serum levels of tumor-specific biomarkers, including CEA, CA27-29, and CA15-3. CEA is an important serum tumor marker that is often elevated in metastatic or recurrent breast cancer. CA15-3 is widely used in clinical practice for breast cancer owing to its non-invasive nature, accessibility, and cost-effectiveness, aiding in real-time diagnosis, disease monitoring, and prognosis across breast cancer stages. CA27-29, an epitope of the MUC1 glycoprotein, is another valuable marker as elevated levels indicate breast cancer recurrence or metastasis, demonstrating higher sensitivity and specificity, especially in advanced or recurrent disease [23,24].

Furthermore, our findings demonstrate that the combination therapy significantly enhanced tumor cell apoptosis in TNBC xenografts, as evidenced by a substantial increase in terminal deoxynucleotidyl transferase dUTP nick-end labeling (TUNEL)-positive cells compared to the TNBC control and monotherapy groups. This marked increase in TUNEL staining indicated enhanced DNA fragmentation, a hallmark of apoptosis, suggesting effective activation of apoptotic pathways [25].

The observed increases in p53 and Bax expression, alongside a decrease in Bcl-2 levels, in response to the rhAPE1/Ref-1 and ASA combination therapy strongly suggest a potent pro-apoptotic effect, primarily via the intrinsic apoptotic pathway. While the individual role of ASA in modulating Bax and Bcl-2 is established [26,27], the combination treatment’s significant impact on p53, a master regulator of apoptosis, points towards a multifaceted mechanism. p53 is crucial in mediating apoptosis in breast cancer by regulating the Bcl-2 family of proteins [28]. Consequently, p53 upregulation may represent an effective strategy for inducing apoptosis in breast cancer treatment [29,30].

RAGE, a multiligand cell-surface, pattern-recognition receptor, has been implicated in cancer progression. The binding of ligands to RAGE activates multiple signaling pathways, resulting in diverse cellular responses depending on the ligand, environment, and cell type. Acetylated APE1/Ref-1 can bind to RAGE [13,15], as a putative binding receptor, and induces apoptosis in ovarian and breast cancer cells [12,13,14]. Therefore, the interaction between acetylated APE1/Ref-1 and RAGE may contribute to the enhanced apoptosis observed with the combination therapy.

The neutrophil-to-lymphocyte ratio (NLR) is a valuable cancer biomarker reflecting the balance between neutrophils and lymphocytes, two crucial components of the immune system. In a breast cancer mouse model, treatment with both rhAPE1/Ref-1 and ASA suppressed tumor growth and reduced NLR, suggesting enhanced immune function and reduced inflammation [31,32]. An elevated NLR has been associated with increased mortality in several malignancies [33,34,35]. Therefore, the observed NLR reduction with the rhAPE1/Ref-1 and ASA treatment may reflect improved prognosis and potential survival benefits.

PTX treatment effectively suppressed tumor growth and induced severe hematological toxicity, including anemia and thrombocytopenia. PTX is known to cause significant myelosuppression, leading to decreased red blood cell and platelet counts [36]. Unlike PTX, which induced severe anemia and thrombocytopenia, the rhAPE1/Ref-1 and ASA combination therapy did not exhibit significant hematological toxicity, suggesting potential safety and tolerability advantages of the combination therapy over conventional chemotherapy agents like PTX. This combination may help reduce the hematological side effects commonly observed with standard chemotherapeutic agents.

Despite the potent synergistic efficacy and favorable safety profile observed in this preclinical TNBC model, several critical challenges must be addressed for the successful clinical translation of the rhAPE1/Ref-1 and ASA combination therapy. A primary concern for any recombinant protein therapeutic approach is pharmacokinetics (PK). As an exogenous protein, systemically administered rhAPE1/Ref-1 is highly susceptible to rapid proteolytic degradation, likely resulting in a short in vivo half-life. This limitation necessitates future research to dedicate efforts to optimizing the protein’s bioavailability. Engineering rhAPE1/Ref-1-Fc fusion proteins is a potential delivery strategy that employ the neonatal Fc receptor pathway to significantly extend serum half-life and improve dosing frequency [37]. Additionally, the long-term safety and potential immunogenicity of rhAPE1/Ref-1 are important considerations. The risk of generating neutralizing antibodies in humans must be evaluated. Such immune responses could not only diminish the therapeutic effect but also lead to adverse immune-related events. Therefore, future preclinical studies are essential to establish the long-term safety profile and confirm the non-immunogenic potential of rhAPE1/Ref-1 before progression to human clinical trials.

Our findings suggest that combining rhAPE1/Ref-1 and ASA represents a promising therapeutic strategy for TNBC. This combination treatment demonstrated synergistic antitumor effects, good tolerability, and modulation of tumor biomarkers, highlighting its potential for clinical translation.

## Figures and Tables

**Table 1:** Changes in hematological parameters following combined ASA and rhAPE1/Ref-1 treatment in TNBC xenograft mice.

Blood Count Parameter	Normal (*n* = 5)	Control (*n* = 4)	rhAPE1/Ref-1 (*n* = 4)	ASA (*n* = 5)	ASA + rhAPE1/Ref-1 (*n* = 4)	PTX (*n* = 4)
Red blood cell (1 × 10^6^ cells/µL)	8.71 ± 0.24	7.62 ± 0.79	7.69 ± 0.46	8.41 ± 0.40	8.19 ± 0.58	1.86 ± 0.62 ***^,###^
Hemoglobin (g/dL)	12.14 ± 0.32	10.53 ± 0.86	10.63 ± 0.52	11.92 ± 0.53	10.93 ± 0.72	2.13 ± 0.72 ***^,###^
Hematocrit (%)	36.66 ± 0.92	32.23 ± 3.13	33.00 ± 1.49	35.88 ± 1.65	33.63 ± 2.38	12.63 ± 4.80 ***^,###^
Platelet (1 × 10^3^ cells/µL)	761.8 ± 95.2	1014.0 ± 144.7	759.0 ± 73.9	983.0 ± 131.4	602.0 ± 46.81	74.33 ± 13.60 ***^,###^
White blood cell (1 × 10^3^ cells/µL)	1.70 ± 0.33	2.56 ± 0.78	1.85 ± 0.32	2.88 ± 0.48	1.46 ± 0.33	1.74 ± 0.33
Monocyte (%)	7.60 ± 1.92	8.23 ± 1.65	8.53 ± 1.12	10.00 ± 1.84	10.13 ± 0.73	8.60 ± 0.56
Eosinophil (%)	6.06 ± 0.92	7.90 ± 2.18	7.13 ± 1.58	3.55 ± 0.78	4.80 ± 0.77	2.97 ± 0.53
Basophil (%)	0.20 ± 0.13	0.00 ± 0.00	0.30 ± 0.11	0.34 ± 0.10	0.00 ± 0.00	0.167 ± 0.08
Neutrophil (%)	35.84 ± 3.73	53.70 ± 4.0 *	47.40 ± 6.5	54.72 ± 3.16 **	39.87 ± 1.61	44.97 ± 2.26
Lymphocyte (%)	54.30 ± 2.17	30.17 ± 3.3 ***	36.63 ± 4.17 ***	29.50 ± 1.56 ***	45.20 ± 2.12 ^##^	43.30 ± 1.26 *^,##^
Neutrophil/Lymphocyte Ratio (NLR)	0.65 ± 0.04	1.96 ± 0.41 **	1.45 ± 0.33	1.94 ± 0.16 ***	0.89 ± 0.08 ^#^	1.05 ± 0.08 *^,#^

Data are presented as mean ± standard error. * *p* < 0.05, ** *p* < 0.01, and *** *p* < 0.001 vs. normal; ^#^ *p* < 0.05, ^##^ *p* < 0.01, and ^###^ *p* < 0.001 vs. control. Normal: BALB/c nude; control: mice implanted with triple-negative breast cancer cells; rhAPE1/Ref-1: mice implanted with triple-negative breast cancer cells and treated with recombinant APE1/Ref-1 protein; ASA: mice implanted with triple-negative breast cancer cells and treated with aspirin; ASA+rhAPE1/Ref-1: mice implanted with triple-negative breast cancer cells and treated with recombinant APE1/Ref-1 protein and aspirin; PTX: mice implanted with triple-negative breast cancer cells and treated with paclitaxel.

## Data Availability

The original contributions presented in this study are included in the article/Supplementary Materials. Further inquiries can be directed to the corresponding authors.

## Supplementary Materials

The following supporting information can be downloaded at: https://www.mdpi.com/article/10.3390/biomedicines13112767/s1. Figure S1: Combined in vitro treatment with acetylsalicylic acid (ASA) and recombinant human APE1/Ref-1 (rhAPE1/Ref-1) reduces cell viability in MDA-MB-468 cells.

## Abbreviations

The following abbreviations are used in this manuscript:

rhAPE1/Ref-1	recombinant human apurinic/apyrimidinic endonuclease 1/redox factor-1
ASA	acetylsalicylic acid
TNBC	triple-negative breast cancer
Ac-APE1/Ref-1	acetylated apurinic/apyrimidinic endonuclease 1/redox factor-1
RAGE	receptor for advanced glycation end-products
Ad-PPTLS-APE1/Ref-1	adenoviral vector expressing PPTLS-tagged APE1/Ref-1
PTX	paclitaxel
CEA	carcinoembryonic antigen
CA27-29	cancer antigen 27-29
CA15-3	cancer antigen 15-3
