# Supplementary material for: Synergistic Inhibition of Triple-Negative Breast Cancer by Acetylsalicylic Acid and Recombinant Human APE1/Ref-1 in a Mouse Xenograft Model
Source: Biomedicines. 2025 Nov 12;13(11):2767. doi: 10.3390/biomedicines13112767 (PMC12650036; doi:10.3390/biomedicines13112767)
Supplement: Supplementary file 1 [file biomedicines-13-02767-s001.zip › biomedicines-3894363-supplementary.pdf]

biomedicines

MDPI

1. Supplementary material

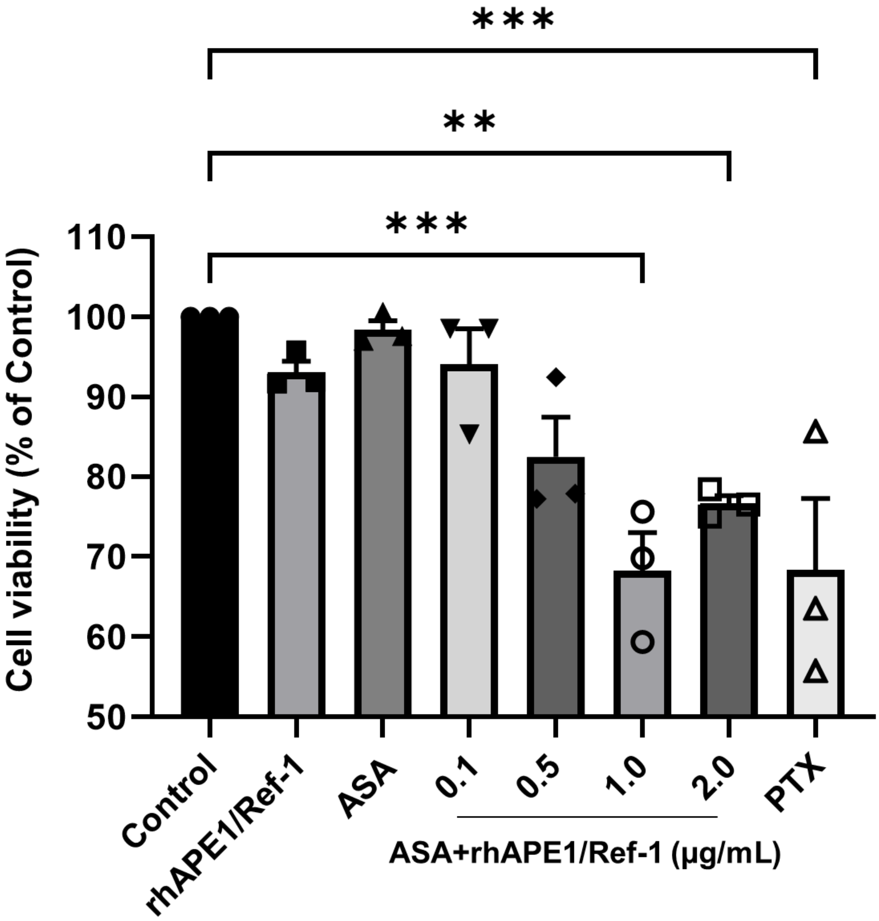

Supplementary Figure S1. Combined in vitro treatment with acetylsalicylic acid (ASA) and recombinant human APE1/Ref-1 (rhAPE1/Ref-1) reduces cell viability in MDA-MB-468 cells. Cell viability of MDA-MB-468 cells was measured with MTT assay following rhAPE1/Ref-1 treatment in the presence or absence of ASA. Paclitaxel (PTX) was used as positive control. Cell viability is expressed as a percentage relative to the control group. Data are presented as the mean ± SE. $^{**}p < 0.01$ and $^{***}p < 0.005$ vs. control.

2. Supplementary method

The human breast adenocarcinoma cell line MDA-MB-468 (ATCC® HTB-132™) was obtained from the American Type Culture Collection (Manassas, VA, USA). The cells were maintained as monolayers in 75-cm^2 tissue culture flasks at 37 °C in a humidified incubator with 5% CO_2.MDA-MB-468 cells were cultured in RPMI-1640 medium (Thermo Fisher Scientific, Waltham, MA, USA) supplemented with 10% heat-inactivated fetal bovine serum and 1% penicillin-streptomycin (Thermo Fisher Scientific). For the cell viability assay, MDA-MB-468 cells were seeded in 96-well plates and treated with recombinant human APE1/Ref-1 (rhAPE1/Ref-1, 0.1 ~ 2 μg/mL), acetylsalicylic acid (ASA, 1mM) or paclitaxel (5 μg/mL) for 24 h. After treatment, 10 μL of MTT reagent (5 mg/mL) was added to each well and incubated for 2 h at 37 °C. The resulting formazan crystals were dissolved in 100 μL of DMSO, and absorbance was measured at 600 nm using a GloMax® Discover Microplate Reader (Promega, Madison, WI, USA).
